# A Novel Quantitative Approach to Women’s Reproductive Strategies

**DOI:** 10.1371/journal.pone.0046760

**Published:** 2012-10-02

**Authors:** Fritha H. Milne, Debra S. Judge

**Affiliations:** School of Anatomy, Physiology and Human Biology, The University of Western Australia, Perth, Western Australia, Australia; German Primate Centre, Germany

## Abstract

The patterned way in which individuals allocate finite resources to various components of reproduction (e.g. mating effort, reproductive timing and parental investment) is described as a reproductive strategy. As energy is limited, trade-offs between and within aspects of reproductive strategies are expected. The first aim of this study was to derive aspects of reproductive strategies using complete reproductive histories from 718 parous Western Australian women. Factor analysis using a subset of these participants resulted in six factors that represented ‘short-term mating strategy’, ‘early onset of sexual activity’, ‘reproductive output’, ‘timing of childbearing’, ‘breastfeeding’, and ‘child spacing’. This factor structure was internally validated by replication using a second independent subset of the data. The second aim of this study examined trade-offs between aspects of reproductive strategies derived from aim one. Factor scores calculated for each woman were incorporated in generalised linear models and interaction terms were employed to examine the effect of mating behaviour on the relationships between reproductive timing, parental investment and overall reproductive success. Early sexual activity correlates with early reproductive onset for women displaying more long-term mating strategies. Women with more short-term mating strategies exhibit a trade-off between child quantity and child quality not observed in women with a long-term mating strategy. However, women with a short-term mating strategy who delay reproductive timing exhibit levels of parental investment (measured as breastfeeding duration per child) similar to that of women with long-term mating strategies. Reproductive delay has fitness costs (fewer births) for women displaying more short-term mating strategies. We provide empirical evidence that reproductive histories of contemporary women reflect aspects of reproductive strategies, and associations between these strategic elements, as predicted from life history theory.

## Introduction

Individuals may exhibit particular strategies to optimize their reproductive output for maximum fitness. Resources (time and energy) are limited and when allocated to one life purpose become unavailable for others [1]. Trade-offs are therefore inevitable, and occur within and between elements of reproductive strategies [2]. Note that the use of the term strategy does not imply a conscious plan, or awareness [3], [4]. Aspects of reproductive strategies include mating effort, timing of reproduction and parental effort [5]. Real life measures of these aspects of reproductive strategies and their outcomes include variables such as age at menarche [6], number of sexual partners (desired [7] or actual [8]), age at first birth [9], [10] and number of children born [8]. Various authors have used, these, and other similar variables, to indicate ‘strategies’ and fitness outcomes. However, to our knowledge, the empirical organisation of reproductive variables into larger strategies has not been demonstrated for humans.

The first aim of this study is therefore to investigate whether reproductive variables are associated in ways that reflect aspects of reproductive strategies for contemporary human females. We hypothesise that reproductive variables will organise into factors that reflect variation in patterns of mating behaviours, reproductive timing, parental investment strategies and reproductive success as predicated by life history theory.

To test aim one we employ factor analysis to deduce aspects of reproductive strategies from the reproductive histories of a sample population of contemporary Western Australian, mostly post-menopausal, women. One advantage of this study is that the majority of participants are post-menopausal; this enables us to investigate complete reproductive histories. Factor analysis is a quantitative and unbiased approach that derives empirical latent factors from many correlated variables [11]. Factor analysis is therefore used when there are multiple interrelated items that theoretically represent an underlying trait or traits [11]. Factor analysis acts as a method for data or variable reduction analyses by concatenating variables into a smaller number of factors. Thereby making further analyses less complex [12]. Researchers in psychology often employ factor analysis to determine how a suite of personality and/or cognitive characteristics fit together to represent underlying (latent) traits [13]–[18]. Despite its common use in psychology, factor analysis has not been used to empirically derive aspects of human reproductive strategies. We believe this methodology will prove to be a useful tool in the field of human behavioural ecology. Firstly, the use of factor analysis will facilitate investigations of the impact of the physical and social environment on reproductive behaviours by decreasing the number of outcome variables requiring separate modelling, thus simplifying further analyses. Secondly, variable reduction facilitates the characterisation of complex life traits and thus is useful in examining life history impacts on adult disease [19] and more readily allows for interdisciplinary research with disciplines such as epidemiology and medical anthropology. Another advantage of the factor analysis approach is that it does not presume what associations exist between the variables, but rather empirically derives a structure based on the data itself.

Two previous studies have investigated the clustering of life-history traits using similar approaches across species of mammals [20], [21]. Stearns [21] used principal components analysis on nine life history traits across two data sets consisting of 65 and 162 mammalian species. After controlling for size and phylogenetic effects, the first principal component displayed a continuum from fast to slow life history strategy; at one end of the factor were small, early maturing, short lived, short gestation, large litter species and at the other were larger species with delayed maturation, long lifespans, long gestation periods and fewer offspring [21]. Biebly et al. [20] performed a similar study using factor analysis with seven variables for 267 mammalian species. In Biebly et al.’s study, after controlling for body size, two factors were extracted. The first represented timing of reproductive bouts and was similar to the fast-slow continuum extracted by Stearns [20]. The second factor represented reproductive output per bout with large litters of small neonates after short gestation at one end and small litters of large neonates after long gestation at the other [20]. These studies indicate that factor analyses can reveal combinations of life-history traits that make theoretical sense, and across species there is a trade-off between maturing and reproducing rapidly versus delaying maturation and reproduction in favour of higher parental investment in fewer children.

Another life history trade-off that has been the subject of much investigation is that of maximising short-term fitness benefits by increasing numbers of children (child quantity) versus maximising long-term fitness benefits by decreasing the intergenerational variance in number of offspring (i.e. to have fewer, higher quality offspring with higher competitive abilities) [22], [23]. In a study of American men and women, individuals coming from larger family sizes had decreased educational attainment [24]. Educational attainment is one measure of child quality [23]; therefore Blake [24] provides evidence that a trade-off between quantity (family size) and quality (educational attainment) exists for contemporary humans. Rather than investigating child quality per se, studies have used measures of parental investment, as a proxy for child quality [25]. Breastfeeding is a common measure of parental investment [26]–[28]. Breastfeeding is associated with child quality outcomes; in the first few months of life infants exclusively breastfed are at a lower risk of death than infants not breastfed [29], [30]. Another measure of parental investment is child spacing [31]. A study of !Kung found that women with longer birth intervals (up to about 72 months) between their children had greater child survival [25]; increasing investment per child increases child survival. Therefore, parental investment measures are useful proxies for child quality.

Parental investment strategies are related to mating strategies in males [32]. Studies of birds [33], non-human primates [34] and humans [35], [36] find that males increase their reproductive success either through investing in higher numbers of mates or through maintaining a monogamous pair-bond with high parental effort. The strategy is dependent on environmental conditions, including resource availability [36], [37]. In a polygynous 19^th^ century human population, poor males (lower quality) with more wives had lower offspring survival [35]. Indicating that under poor conditions, males that shift to maintaining fewer pair-bonds with higher parental investment increase reproductive success [35]. Although most of the work investigating the variation in mating effort has focussed on males, we predict that similar variation in mating behaviour will be evident for human females.

The second aim of this study is to investigate relationships between the reproductive factors extracted from part one. We hypothesise that they will reflect trade-offs predicted by life history theory. We expect human females to exhibit evidence of two major life history trade-offs. Firstly, based on the work of Stearns [21] and Bielby et al. [20] we predict that early maturing, early reproducing individuals will be differentiated from individuals with delayed maturation and reproductive timing. Secondly, we predict evidence of a trade-off between quantity (reproductive output) and quality of offspring. We use breastfeeding per child (parental investment measure) as a proxy for child quality. Thirdly, given empirical validation of variation in female mating effort (as is predicted), we investigate the effect of this variation in mating behaviour on current versus future reproduction, quantity versus quality of offspring, and overall reproductive success. We investigate these relationships using post-reproductive women from a contemporary Western population.

## Methods

Women were recruited, via the Western Australian Cancer Registry, to participate in a larger project concerning the influence of the childhood environment and adult reproductive behaviours on the risk of developing endometrial cancer. The selection criteria for participants included women who were diagnosed with cancer from 2000 to 2009 (inclusive). Three thousand and three women were invited to complete a questionnaire, which asked about their early childhood environment, reproductive histories and adult lifestyle. One thousand, one hundred and ninety two women returned completed questionnaires. After accounting for women who did not receive questionnaires (e.g. died before receiving questionnaire or the questionnaire pack was returned to sender) or who were not eligible to participate (e.g. had cervical cancer, wrongly diagnosed with cancer, wrongly coded as female, or did not speak English) the overall response rate overall was 42.1% (1192/2833). Given the average age of the sample (older people exhibit lower response rates [38], [39]), the length of the questionnaire (longer questionnaires have lower response rates [40]), and the potentially sensitive questions addressed in the questionnaire itself, this is a reasonable response rate. The Australian National Endometrial Cancer study had a similar response rate of 54.4% [41]. Respondents did not differ from non-respondents in terms of year of cancer diagnosis (χ^2^_(1)_ = 1.887, p = 0.170) or postcode at cancer diagnosis (χ^2^_(1)_ = 1.307, p = 0.253). As expected, respondents were significantly younger than non-respondents (χ^2^_(1)_ = 14.961, p<0.001). Finally, given that non-responders do not represent a homogenous group [42] it is unlikely that non-responders would significantly differ from responders in any reproductive behaviour questions.

Women were excluded if more than 50% of their reproductive history data were missing (n = 42), if they were nulliparous (n = 92), if they reported having had a pre-menopausal hysterectomy (n = 283) or if they had cancer types not specified by the selection criteria (n = 57). Although it would be interesting to include nulliparous women, due to the small numbers (n = 92), and the heterogeneous nature of this group (including 15 women who never had sexual intercourse, 48 women who had sex but never got pregnant and 29 who became pregnant but never gave birth), we decided to exclude them from these analyses. This resulted in a final sample of 718 parous women. Seventy percent of women had no missing reproductive data, and only 1% of women had missing information for 31–50% of their reproductive variables. Missing values analysis, using maximum likelihood estimation, in SPSS v18 was used to calculate missing values for the reproductive variables. To check the impact of missing values analysis the same analyses were run excluding women with any imputation of data and the results did not differ (Table S1).

There were initially 13 reproductive variables of interest (Table 1). Women were asked to provide their age at first menstrual period (menarche) and age at first consensual sexual intercourse with a male partner. Number of sexual partners was collected as a categorical variable to maximise response rate for this potentially sensitive question. The categories for number of sexual partners were 1–2 (reference category), 3–4, 5–9, and 10+ (Table 1). Numbers of (committed sexual) relationships, pregnancies and children were collected as open-ended continuous variables. Due to the small proportion of women with extreme values, upper values were truncated into one category (Table 1). A committed sexual relationship was defined as one that lasted more than 6 months, or where the couple had cohabited, or been engaged or married. Women were asked to provide the total number of years in which they were involved in committed sexual relationships. By dividing this value by the number of committed sexual relationships, each woman’s average duration of committed relationships (in years) was calculated. Ages at first and last birth were calculated from the difference between the woman’s age at participation and the month and year of birth of her first and last child, respectively. Ever breastfed was a dichotomous variable; 86.1% of women had breastfed at least one child (Table 1). Never breastfed was the reference category. Total duration of breastfeeding was calculated by summing the number of months a woman reported having breastfed each child. Results did not differ if average duration of breastfeeding per child was employed instead (Table S2). Menopause was defined as reported age at the earliest of natural menopause, double oophorectomy, or hysterectomy. Results did not differ if only women with natural menopause were included (Table S3). Average inter-birth interval (IBI) was calculated by taking the age difference between the first and last child and dividing it by the number of children less one. Inter-birth interval is a proxy for investment in each child [2]; IBI is the period during which a child has no younger competitors and constant investment, thus ‘only’ or sole children have a very long period of uninterrupted investment. Ten percent (n = 74) of women were uniparous and we assigned them an average IBI of five. Fourteen women (19% of the uniparous women) were under 50 years of age, and could potentially have future children. The assigned value of five years is greater than the average IBI (91^st^ percentile) without being an extreme value (maximum = 19.5 years). In addition, the same analyses were performed setting IBI for uniparous women to the difference between their current age and age at birth for pre-menopausal women, or the difference between their age at menopause and age at birth for postmenopausal women with no significant variation in results (Table S4). The results also did not differ if only women with two or more children were included (Table S5). Therefore, five is a conservative imputed value for IBI that does not disproportionately influence the model.

**Table 1:** Descriptive statistics for the reproductive variables investigated.

Variable Name (continuous variables a)	Mean ± std dev	Min - Max
*Age at menarche*	12.94±1.354	9.0–17.0
*Age at first sexual intercourse*	19.69±3.326	12.0–36.0
*Number of committed relationships*	1.7±1.4	1–20^c^
*Average duration of committed relationship (years)*	30.23±15.659	1.0–65.0
*Number of pregnancies*	3.3±1.54	1–13^d^
*Age at first birth*	25.09±4.895	15.1–44.3
*Age at last birth*	30.51±5.053	16.8–50.9
*Number of children*	2.7±1.17	1–9^e^
*Average inter-birth interval (years)*	3.35±1.879	0.7–19.5
*Total duration of breastfeeding across all children (months)*	14.31±15.313	0.0–153.0
*Age at menopause* (for 659 post-menopausal women)	49.45±4.613	30.0–60.0
**Variable Name (categorical variables** b **)**	**Level Name**	**Percent**
*Number of sexual partners*	1–2	62.7
	3–4	19.4
	5–9	11.8
	10+	6.1
*Ever breastfed a child*	No	13.9
	Yes	86.1

a For continuous variables the mean, standard deviation, minimum and maximum score are provided.

b For categorical variables the percent of women in each level of the variable are provided.

c Cases with 4+ committed relationships aggregated for analyses (8.5%).

d Cases with 6+ pregnancies aggregated for analyses (8.8%).

e Cases with 5+ children aggregated for analyses (6.1%).

All summary statistics are for the full 718 women (unless otherwise stated).

The cases were split into two halves using the ‘split file at random’ command in SPSS v18. Both data subsets had similar numbers of pre and post-menopausal women, women with only one child, and variety of cancer types. Splitting the data allowed us to develop a model on the first data subset, and to then test that model for internal validity on the second data subset.

To test aim one, exploratory factor analysis with an oblique rotation (oblimin) was employed using Mplus v6.11. Oblique rotation produces a simple structure which facilitates factor interpretation, and allows the factors to be correlated [43]. Mplus allows the inclusion of continuous and non-continuous variables in factor analyses.

Exploratory factor analysis was run on the first data subset. A range of factor structures was tested (including between one and thirteen factors), and a series of fit statistics were used to determine which model exhibited good fit. The *Chi-squared test of model fit* tests for a difference between the variance-covariance matrix of the data and the variance-covariance matrix estimated by the model [44]; a good model will show no significant difference. *Pclose* is the significance test of whether the *Root Mean-Square Error of Approximation* (RMSEA) differs from 0 [45]; *Pclose* should be non-significant (i.e. no error). The *Tucker-Lewis Index* (TLI) and the *Comparative Fit Index* (CFI) measure the fitted model compared to a baseline model (usually the null or independence model) [45]; the closer the value to 1 the better the fitted model. Once good fit was established, the final model was forced onto the second data subset and the above parameters were examined to test the model for internal validity.

To test aim two, a score for each participant for each factor (factor scores) was calculated. To do this the internally validated final model was forced onto the full data set. The factor loadings from the full data set were then used to calculate individual factor scores using the regression method known as the modal posterior estimator [46], [47].

Generalised linear models were employed to investigate the relationships between factors extracted from aim one and additional covariates. These covariates included age, natal family socioeconomic status (FSES; three levelled categorical variable; poorer than most (reference category), of average wealth, wealthier than most), and educational attainment (EDUC; five levelled categorical variable; did not complete year 10 (reference category), year 10 completed, upper high school completed, TAFE or trade completed, university of college graduate). Variation in mating effort was investigated first to see what characteristics influenced a woman’s mating behaviour. Then, factors influencing reproductive timing, parental investment strategies and reproductive success were investigated. Interaction terms were employed to see whether mating behaviour altered the relationships between the factors.

### Ethics Statement

The Western Australian Department of Health and the University of Western Australia Human Research Ethics Committees approved this research. Completion and return of the questionnaire demonstrated implied consent as specified in the information letter, thus allowing the questionnaire responses to remain anonymous and confidential.

## Results

Women averaged 65 years of age (range = 34–95 years) and 91.7% were postmenopausal. Therefore, the vast majority of women had completed their reproductive careers. Additionally, 85% of the women were diagnosed after 50 years of age; thus, it is unlikely that the cancer diagnosis altered the majority of the women’s reproductive trajectories. Of the 120 women diagnosed with cancer before age 50, only 24 (3.3% of 718) had a gynaecological cancer. Cancer diagnoses are unlikely to have had an influence on reproductive histories.

Eighty one percent of women in this sample reported having used contraceptives (including barrier methods, IUDs, hormonal pills, injections and implants) at some point during their lives. The availability and use of contraception may sever evolutionary linkages between mating behaviours and reproductive output [3], [48]. However, markers of reproductive behaviours that precede conception (e.g. age at first sexual intercourse) are expected, even in contemporary populations, to exhibit differences that represent differing reproductive strategies or elements of reproductive strategies [3].

### Development of the Model on the First Data Subset

Initial factor analysis of the first data subset resulted in the successive exclusion of two reproductive variables due to bad fit statistics and low communalities; they were menarche (communality = 0.016 or 1.6%) and menopause (communality = 0.032 or 3.2%). Communalities are the percent of variance in each measured variable explained by the factor structure [49]. Variables with low communalities do not associate with the other variables in a factor structure even though they may be important components of a life history. Communalities for all other variables ranged from 0.536 to 0.992.

After exclusion of these two variables, a second exploratory factor analysis on the first half of the data was run using the remaining eleven variables. Factor structures with one to eleven factors were tested and the six-factor model was the most parsimonious factor structure to exhibit good fit (χ^2^_(4)_ = 4.426, p = 0.351, RMSEA = 0.017 (90% CI = 0.000–0.083), PCLOSE = 0.717, CFI = 1.000, TLI = 0.998). The communality for each item ranged from 0.589–0.987, with an average of 0.845.

### Testing Internal Validity with Second Data Subset

Subjecting the second data subset to an exploratory factor analysis with a six-factor set with the eleven variables included above also resulted in good fit statistics (χ^2^_(4)_ = 6.692, p = 0.153, RMSEA = 0.043 (90% CI = 0.000–0.099), PCLOSE = 0.499, CFI = 0.999, TLI = 0.989). The communalities for all items ranged between 0.694–0.990, with an average of 0.879.

The factor loadings from the model on both the first and second data subsets were very similar (Table S6). When comparing the same factor across the two models, the magnitude of the factor loadings is important but a consistent change in the sign of the loadings is not. Factor loadings reflect the correlation between a factor and a variable [43]. Factors 3–6 are almost identical across the two subsets (Table S6). Factors 1 and 2 differed somewhat; number of sexual partners clustered with number of committed relationships and average duration of relationships in the first data subset; however, in the second data subset, number of sexual partners clustered with age at first sexual intercourse (Table S6). Given the random split of the cases, the strong correspondence between analyses indicates a very robust factor structure with high internal validity.

### Calculating Factor Scores on the Full Data Set

A factor analysis on the full data set with a 6-factor constraint was used to enable the calculation of factor scores for all individuals for use in further analyses. The full model retained good fit statistics (χ^2^_(4)_ = 3.942, p = 0.414, RMSEA = 0.000 (90% CI = 0.000–0.056), PCLOSE = 0.914, CFI = 1.000, TLI = 1.000). The communalities ranged from 0.682–0.989, with an average of 0.888.

After reviewing the factor loadings from the full data set (Table 2), labels were assigned to each of the factors representing the variables with high factor loadings. Factor labels are indicated by the use of single quotes. Factor 1 loaded positively on number of committed relationships, and negatively on average duration of committed relationships (Table 2). Therefore, at high score end of the factor 1 continuum were short-term mating strategists with more partners and shorter durations of relationships, while the low score end was characterized by long-term mating strategists with fewer partners and relationships of longer average duration; thus, we label factor 1 ‘short-term mating strategy’. Factor 2 loaded most highly on age at first sexual intercourse (Table 2) and was labelled ‘early onset of sexual activity’. Note that because of the negative factor loading, a woman with a high score on ‘early onset of sexual activity’ displays a young age at first sexual intercourse, while a low score for ‘early onset of sexual activity’ represents an older age at first sexual intercourse. Factor 3 loaded most highly on number of pregnancies and number of children (Table 2), and was labelled ‘reproductive output’. Ages at first and last births clustered together on factor 4 (Table 2). Factor 4 was labelled ‘timing of childbearing’. Breastfeeding experience and total duration of breastfeeding clustered together on factor 5 (Table 2), which was labelled ‘breastfeeding’. Factor 6 loaded most highly on average inter-birth interval (Table 2) and was labelled ‘child spacing’.

**Table 2:** Pattern matrix with rotated factor loadings for each variable in the six-factor structure on the full data subset.

	1	2	3	4	5	6
	Short-term mating strategy	Early onset of Sexual Activity	Reproductive output	Timing of childbearing	Breastfeeding	Child Spacing
*Age at first sexual intercourse*	−0.041	−**0.727**	−0.070	0.202	−0.011	−0.031
*Number of sexual partners*	0.482	0.489	−0.151	0.203	0.005	0.004
*Number of committed relationships*	**0.904**	0.152	0.076	−0.003	−0.044	0.002
*Average duration of relationships*	−**1.011**	0.116	0.033	0.031	−0.028	−0.014
*Number of pregnancies*	−0.021	0.191	**0.780**	0.112	0.056	−0.049
*Age at first birth*	0.031	−0.084	−0.399	**0.835**	0.010	−0.214
*Age at last birth*	−0.004	−0.054	0.298	**0.906**	0.021	0.232
*Number of children*	−0.018	−0.057	**1.040**	−0.030	0.046	−0.091
*Average inter-birth interval*	0.022	0.010	−0.148	0.022	0.003	**0.945**
*Ever breastfed*	−0.014	0.029	−0.086	−0.072	**1.016**	−0.041
*Duration of breastfeeding*	0.017	−0.030	0.121	0.087	**0.940**	0.055

These factor loadings are the ones employed to calculate the factor scores for each woman. They provide the direction and magnitude of the relationship between each variable and factor.

Bolding shows factor loadings above |0.5|.

### Aim 2: Investigating Relationships between Extracted Reproductive Factors

‘Early onset of sexual activity,’ age at menarche, FSES and age were regressed on ‘short-term mating strategy’ to investigate what characteristics influence women’s mating behaviour. After backwards variable selection the final model included:
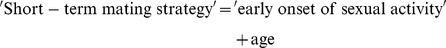



With an increasing score on ‘early onset of sexual activity’ there was an increase in short-term mating strategy (Table 3). With increasing age there was a decrease in short-term mating strategy (Table 3).

**Table 3:** Parameter coefficients and corresponding significance level for each input variable in the final regression models for ‘short-term mating strategy,’ ‘timing of childbearing,’ average duration of breastfeeding per child and ‘reproductive output.’

Outcome variable	Input variable	Parameter coefficient	p-value
*‘Short-term mating strategy’*	*Age*	−0.377	<0.001
	*‘Early onset of sexual activity’*	0.670	<0.001
*‘Timing of childbearing’*	*EDUC1: Did not complete year 10*	0	–a
	*EDUC2: Year 10 completed*	0.318	0.494
	*EDUC3: Upper high school completed*	1.722	0.001
	*EDUC4: TAFE or trade completed*	1.878	<0.001
	*EDUC5: University or college graduate*	3.492	<0.001
	*‘Early onset of sexual activity’*	−0.267	<0.001
	*‘Short-term mating strategy’*	0.034	0.016
	*‘Short-term mating strategy’ x ‘Early onset of sexual activity’*	0.021	<0.001
*Average duration of breastfeeding per child*	*EDUC1: Did not complete year 10*	0	–a
	*EDUC2: Year 10 completed*	−0.065	0.911
	*EDUC3: Upper high school completed*	0.467	0.463
	*EDUC4: TAFE or trade completed*	0.471	0.449
	*EDUC5: University or college graduate*	1.435	0.025
	*Age*	−0.084	<0.001
	*‘Reproductive output’*	−0.094	0.094
	*‘Timing of childbearing’*	0.164	<0.001
	*‘Short-term mating strategy’*	0.000	0.982
	*‘Short-term mating strategy’ x ‘Reproductive output’*	−0.011	0.019
	*‘Short-term mating strategy’ x ‘Timing of childbearing’*	0.007	0.049
*‘Reproductive output’*	*EDUC1: Did not complete year 10*	0	–a
	*EDUC2: Year 10 completed*	−0.646	0.095
	*EDUC3: Upper high school completed*	−0.705	0.097
	*EDUC4: TAFE or trade completed*	−1.124	0.007
	*EDUC5: University or college graduate*	−1.302	0.002
	*Age*	0.067	<0.001
	*‘Timing of childbearing’*	−0.096	0.001
	*‘Short-term mating strategy’*	−0.014	0.257
	*‘Short-term mating strategy’ x ‘Timing of childbearing’*	−0.004	0.086

Educational attainment (EDUC) is a categorical variable. The parameter coefficients and significance levels for each level (1–5) of EDUC are provided.

a EDUC1 is the reference category, thus no p-value can be provided.

**Figure 1:**
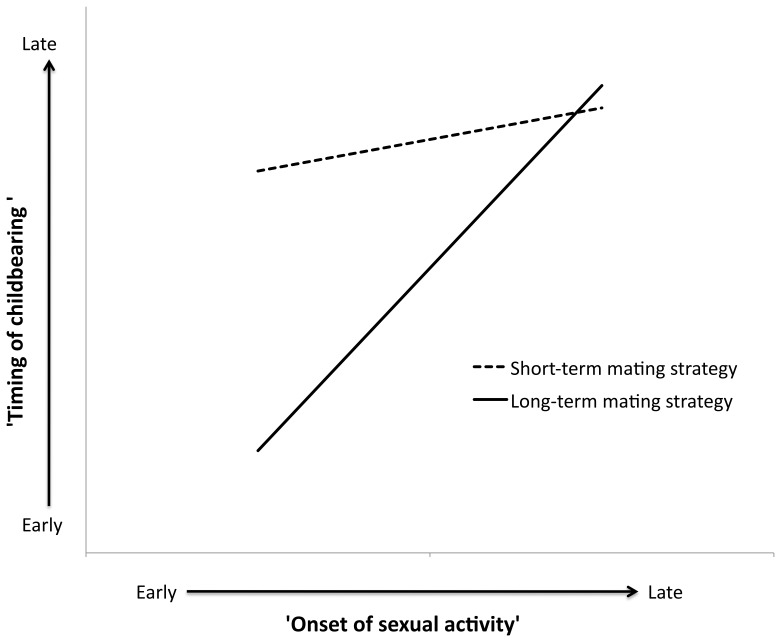
Heuristic diagram of the significant interaction effect of ‘short-term mating strategy’ and ‘early onset of sexual activity’ on ‘timing of childbearing.’ End points calculated using 25^th^ and 75^th^ percentiles for ‘early onset of sexual activity’ and ‘short-term mating strategy,’ with educational attainment at the median (upper high school).

‘Early onset of sexual activity,’ ‘short-term mating strategy,’ age, FSES, educational attainment and an interaction between ‘short-term mating strategy’ and ‘early onset of sexual activity’ were regressed on ‘timing of childbearing’ to investigate the slow-fast continuum, indicating variation in reproductive timing. An interaction term between ‘short-term mating strategy’ and ‘early onset of sexual activity’ was included in order to determine if the association between timing of sexual activity and ‘timing of childbearing’ differed by mating behaviour. After backwards variable selection the final model was:
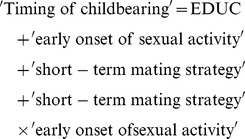



Women with upper high school, TAFE/trade or university/college education had higher scores for ‘timing of childbearing’ compared to women who did not complete year 10 (Table 3). The interaction term for ‘short-term mating strategy’ and ‘early onset of sexual activity’ was significant (Table 3). With delayed timing of sexual activity there was only a slight delay in ‘timing of childbearing’ for women who displayed a more short-term mating strategy, whilst for those with a more long-term mating strategy, a delay in timing of sexual activity resulted in a steep delay in ‘timing of childbearing’ (Figure 1).

**Figure 2:**
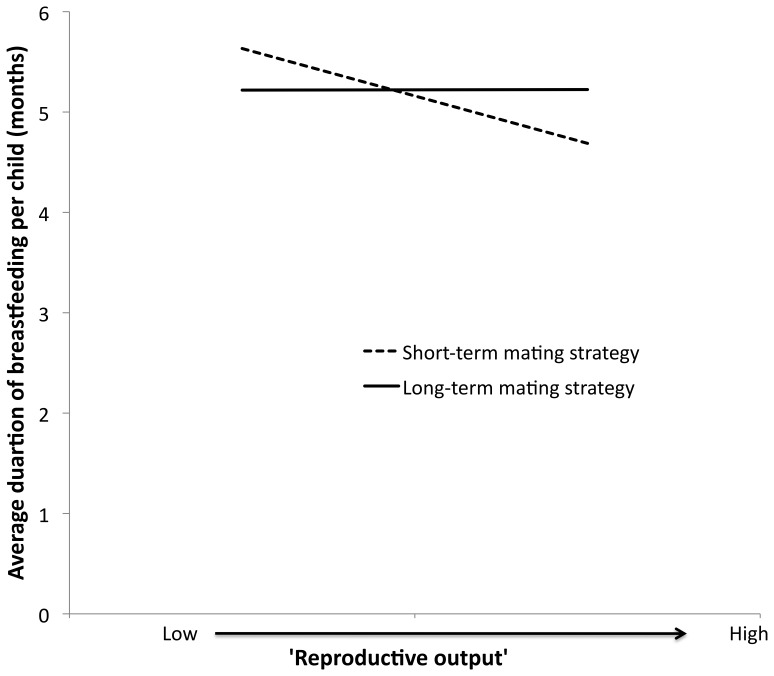
Heuristic diagram of the significant interaction effect of ‘short-term mating strategy’ and ‘reproductive output’ on average duration of breastfeeding per child (in months). End points calculated using 25^th^ and 75^th^ percentiles for ‘reproductive output’ and ‘short-term mating strategy,’ with educational attainment at the median (upper high school), age at the mean (64.44 years) and ‘timing of childbearing’ at the mean.

To investigate parental investment strategies and evidence of a child quantity – quality trade-off in this population of adult women we used the variable average duration of breastfeeding per child. This variable was created by dividing the total duration of breastfeeding across all children by the number of children. ‘Reproductive output’, ‘timing of childbearing,’ ‘short-term mating strategy,’ FSES, educational attainment, age and an interaction between ‘short-term mating strategy’ and ‘reproductive output’ were regressed on average duration of breastfeeding per child. After backwards variable selection the final model was:
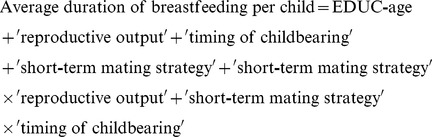



Women with university/college education had higher average duration of breastfeeding compared to women who did not complete year 10 (Table 3). With increasing age, average duration of breastfeeding per child decreased (Table 3). The interaction term ‘short-term mating strategy’ by ‘reproductive output’ had a significant effect on average breastfeeding duration per child (Table 3). For women who displayed a more long-term mating strategy, an increase in ‘reproductive output’ did not influence average breastfeeding duration per child, whilst for women with a more short-term mating strategy an increase in ‘reproductive output’ resulted in decreased average duration of breastfeeding per child (Figure 2).

**Figure 3:**
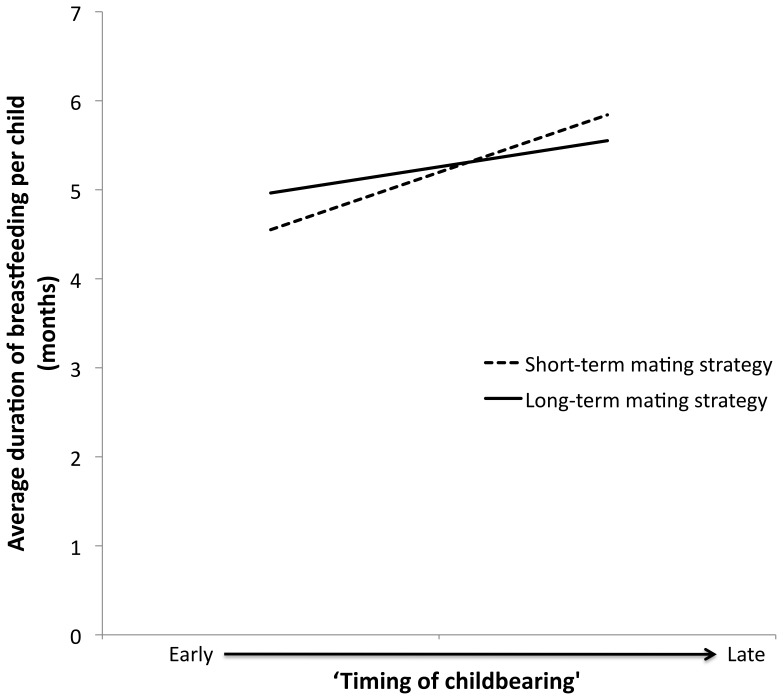
Heuristic diagram of the significant interaction effect of ‘short-term mating strategy’ and ‘timing of childbearing’ on average duration of breastfeeding per child (in months). End points calculated using 25^th^ and 75^th^ percentiles for ‘timing of childbearing’ and ‘short-term mating strategy,’ with educational attainment at the median (upper high school), age at the mean (64.44 years) and ‘reproductive output’ at the mean.

The interaction term ‘short-term mating strategy’ by ‘timing of childbearing’ had a significant effect on average breastfeeding duration per child (Table 3). For women with a more long-term mating strategy there was a marginal increase in breastfeeding duration per child with delayed ‘timing of childbearing’ (Figure 3). However, women with a more short-term mating strategy who delayed ‘timing of childbearing,’ exhibited breastfeeding per child time that approximated or surpassed that of women who exhibited a more long-term mating strategy (Figure 3).

**Figure 4:**
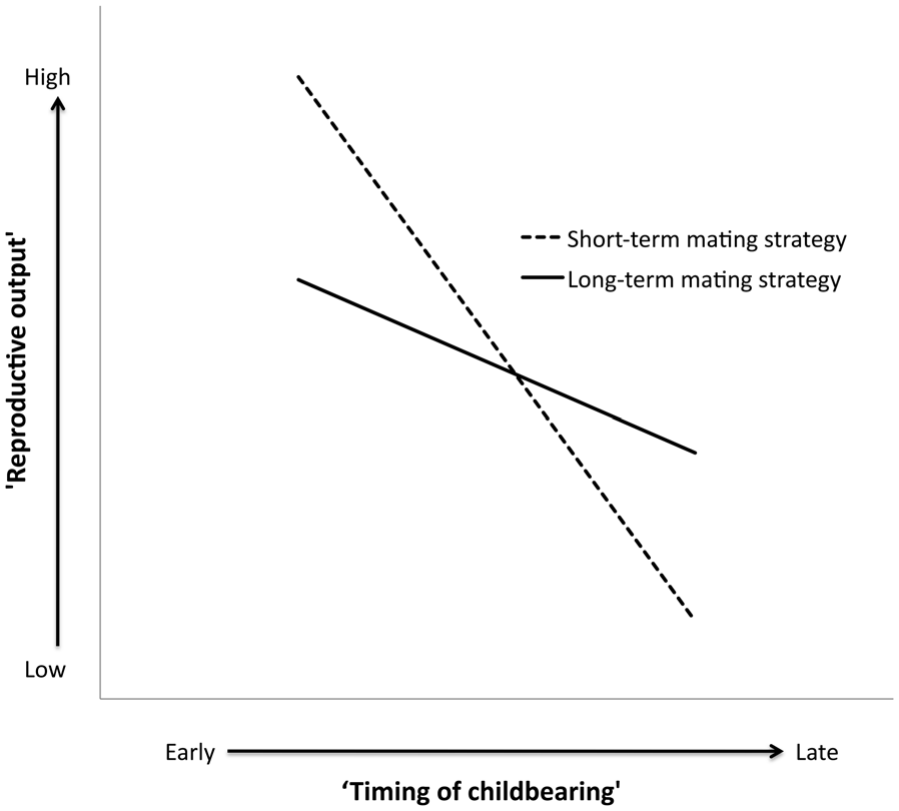
Heuristic diagram of the trend significance interaction effect of ‘short-term mating strategy’ and ‘timing of childbearing’ on ‘reproductive output.’ End points calculated using 25^th^ and 75^th^ percentiles for ‘timing of childbearing’ and ‘short-term mating strategy,’ with educational attainment at the median (upper high school) and age at the mean (64.44 years).

‘Timing of childbearing,’ ‘short-term mating strategy,’ age, FSES, educational attainment and an interaction between ‘timing of childbearing’ and ‘short-term mating strategy’ was regressed on ‘reproductive output’ to investigate which characteristics influenced overall reproductive success (children born). After backwards variable selection the final model was:
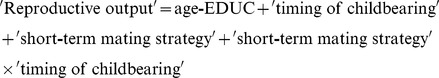



Women with TAFE/trade or university/college education had lower ‘reproductive output’ than women who did not complete year 10 (Table 3). Older women had higher ‘reproductive output’ (Table 3). There was a trend for the interaction term between ‘timing of childbearing’ and ‘short-term mating strategy’ (Table 3). For women with a more long-term mating strategy a delay in ‘timing of childbearing’ resulted in only a slight decrease in ‘reproductive output’, whilst for women with a more short-term mating strategy a delay in ‘timing of childbearing’ resulted in a steep decrease in ‘reproductive output’ (Figure 4).

## Discussion

Life history theory predicts suites of reproductive parameters that are expected to correlate in predictable ways depending on environmental conditions. Relationships between specific life history parameters have been investigated across many species [20], [21], [50], [51]; however, the use of factor analysis to explore the relationships between larger sets of reproductive variables has not been reported for humans. The first aim of this study was to employ exploratory factor analysis to demonstrate that quantitative demographic parameters would reflect latent factors relevant to life history predictions contemporary Western in post-menopausal human females. As expected the six factors derived here delineate aspects of reproductive strategies including mating effort (‘short-term mating strategy’), timing of reproduction (‘early onset of sexual activity’ and ‘timing of childbearing’), parental investment strategies (‘breastfeeding’ and ‘child spacing’) and reproductive success (‘reproductive output’).

Unexpectedly, menarche was not associated with other variables characterising timing of life history strategy and was dropped from the analyses, as was menopause. Therefore, these are not good indicators for differentiation of reproductive strategies for a contemporary population of post-menopausal women. Women in contemporary populations have greater control over the timing of onset of child bearing [10] and age at physiological sexual maturation is not correlated with reproductive timing in this population of women. In a different sample from a similar contemporary Australian population, Milne & Judge [52] found that family composition was correlated with menarche, but not with reproductive onset – revealing a limited association between characteristics influencing both sexual maturation and reproductive onset. On the other hand, a study that investigated women from 22 subsistence-based traditional populations found that age at menarche was strongly and positively correlated with age at first reproduction [53]. Perhaps delayed reproductive timing as a result of increased resource-acquisition time seen in contemporary populations [10] separates the association between sexual maturation and reproductive onset in contemporary populations.

The two proxy measures of parental investment (breastfeeding and inter-birth interval) were not associated. In traditional non-contracepting populations, breastfeeding is the primary mechanism of birth spacing [54] and thus it would be expected that breastfeeding and inter-birth interval cluster together on one factor. Here, the separation of these denotes a disengagement of biological spacing mechanisms in this post-industrial population. Access to contraception may have interrupted the relationship between child spacing and breastfeeding [54], [55].

### Aim 2: Investigating Relationships between Extracted Reproductive Factors

Covariates, including educational attainment and age, influence the reproductive factors in predictable ways. With increasing educational attainment women displayed delayed ‘timing of childbearing,’ reduced ‘reproductive output’ and greater duration of breastfeeding (parental investment) in each child. In developed populations, the cost of reproduction is high [56] and higher education is associated with higher income [57], therefore more educated women may be delaying reproduction to employ a resource-acquisition strategy before commencing reproduction [10]. Additionally, more educated women have been shown to have fewer children than less educated women [57]. As these more educated women are also delaying reproduction it may be that they are employing a child quality strategy. This is supported by our finding that more educated women had longer duration of breastfeeding per child. This result concurs with other studies [58]–[61]. As is expected, this population of women also shows a cohort effect on reproductive output; older women have greater ‘reproductive output’ than younger women. The fertility rate in Australia has dropped from 3.5 babies born per woman in 1980 to 1.7 in 2001 [62]. As expected [63], there is evidence of a secular increase in breastfeeding duration; older women exhibited shorter breastfeeding duration per child.

This analysis indicates that women show similar mating strategies to those previously documented in men [35], [36]. Some women invest more time and/or energy in finding more mates and others invest in maintaining long-term stable pair bonds. We do not have data on partner selection, and thus are not able to investigate whether women demonstrating different mating behaviour also varied in processes or success in mate choice. Future research might profitably delve into relationships between processes of mate choice, success therein, and mating behaviour. There are two potential hypotheses as to why some women employ short-term mating strategies [64]. The first suggests that a short-term mating strategy results from a failure to develop secure attachment during childhood [65] and is a result of personality and attachment problems – i.e. is not adaptive [64] but rather a failed long-term strategy. The second hypothesis suggests that short-term mating strategy is adaptive under unstable, high mortality childhood environmental conditions [3], [66]. Under both hypotheses short-term mating behaviours are associated with stressful childhood environments. Unfortunately at this time we were unable to investigate the effect of early childhood environmental stress directly, but we did have a measure of family socioeconomic status that may act as a proxy for childhood stress. Future work will reveal more about the effect of actual childhood stress measures on reproductive behaviours including mating strategy. Belsky, Steinberg and Draper [3] suggest that through the influence of the childhood environment some individuals will reach sexual maturity earlier and develop behaviour patterns that include early onset of sexual activity and short-term pair bonds. Therefore, we also investigated the effect of age at menarche and timing of sexual activity on mating behaviour. Neither age at menarche nor FSES influenced ‘short-term mating strategy’. Menarche is a physiological event, which is influenced by resource availability [67], and in this resource rich population there may not be enough variation in age at menarche for it to exhibit an association with mating behaviours. We predict that in traditional, non-contracepting populations that menarche would be associated with mating behaviours. Onset of sexual activity, on the other hand, is a behavioural event, and was associated with mating behaviours, in the direction predicted by life-history theory [3], [68], [69], even in this contemporary population.

‘Early onset of sexual activity’ resulted in early ‘timing of childbearing’ for women who display a more long-term mating strategy, but not for those who display a more short-term mating strategy (Figure 1). During the years in which the majority of these women were reproductively active (∼1950–1990), condom usage was low [48]. The contraceptive pill was introduced in 1961 and had a relatively high uptake [48]. The *availability* of contraception is likely to have been the same for all women, regardless of mating strategy. However, it is possible that *use* of contraceptives differed for short-term and long-term mating strategists. The state of being in a committed sexual relationship may be a signal to women employing more long-term mating strategies to begin reproduction whereas women employing more short-term mating strategies, who do not engage in these long-term committed relationships, and do not receive this signal, are more likely to use contraception. Alternatively, the observed differences between short-term and long-term mating strategies may be due to differential termination of early pregnancies. Women with more short-term and long-term mating strategies did not differ in their number of pregnancies (mean number of pregnancies = 3.25±1.6 and 3.38±1.5 respectively), but women employing more short-term mating strategies gave birth to significantly fewer children than those employing more long-term mating strategies (mean number of children = 2.47±1.1 and 2.98±1.2 respectively) – suggesting that women displaying more short-term mating strategies had more disrupted pregnancies than did those with a long-term mating strategy. However, if women with more short-term mating strategies were more likely to terminate early pregnancies, then no association between timing of sexual activity and reproductive onset would be expected for this group. Terminations could be elective or physiological; however, we are unable to tease apart mechanisms with present data. This is, of course, speculative but suggests interesting directions for future research.

Women employing more short-term mating strategies exhibited evidence of a trade-off between child quantity and investment, while those employing more long-term mating strategies did not (Figure 2). Women with more long-term mating strategies had 0.5 more children than those employing more short-term mating strategies and did not reduce investment in breastfeeding as number of children increased. Lack of a trade-off for women with more long-term mating strategies may be due to the presence of a stable partner. A partner’s investment in mother, baby and/or other existing children may enable the mother to invest more time in breastfeeding the newest baby, without cost to existing or future children, particularly within parities exhibited in this low fertility population. This warrants further research to test for the underlying mechanism(s).

Women employing a more short-term mating strategy who delayed reproduction exhibited breastfeeding investment similar to that shown by women with a more long-term mating strategy (Figure 3). There is evidence of a relationship between stressful childhood conditions and short-term, early reproductive strategy [70]–[72]. That delay causes shifts in investment suggests that an intergenerational cycle can be broken if women environmentally inclined to early reproduction are able to delay.

Delayed ‘timing of childbearing’ resulted in decreased ‘reproductive output’ for women employing a more short-term mating strategy, but not for those employing a more long-term mating strategy (Figure 4). Regardless of timing of reproductive onset, women with a more long-term mating strategy who have stable pair bonds, may have enough reproductive lifespan left to have as many children as desired, when average (desired) levels are low in the population [73]. Reproductive timing is disconnected from reproductive output for women with more long-term mating strategies in this low fertility population. However, women with more short-term strategies, by definition, do not form long-term stable pair bonds, and therefore, those who delay reproduction use more time in mating effort and have less time for parental effort.

In summary, the exploratory factor analysis empirically derives factors representing elements of reproductive strategies from reproductive histories of a sample of contemporary women and validates applicability of the concepts of reproductive strategies discussed in human life history theory literature [2] to a contemporary western population. The relationships among factor scores indicate trade-offs between latent characteristics of reproductive strategies. Mating strategies are associated with different patterns of reproduction. Women who display more short-term mating behaviours reproduce early, invest less and have lower numbers of births. Women who display more long-term mating behaviours have higher reproductive output and show no negative impact of these higher numbers of children on investment per child as measured by breastfeeding.

## Supporting Information

Table S1 **Pattern matrix with rotated factor loadings for each variable in the six-factor structure on the data subset with no imputation of missing values.**
(DOC)

Table S2 **Pattern matrix with rotated factor loadings for each variable in the six-factor structure on the data subset using average duration of breastfeeding per child rather than total duration of breastfeeding across all children.**
(DOC)

Table S3 **Pattern matrix with rotated factor loadings for each variable in the six-factor structure on the data subset with only women who had natural menopause.**
(DOC)

Table S4 **Pattern matrix with rotated factor loadings for each variable in the six-factor structure on the data subset with average inter-birth intervals for uniparous women set at the difference between their age at last birth and their age at menopause (or current age if pre-menopausal).**
(DOC)

Table S5 **Pattern matrix with rotated factor loadings for each variable in the six-factor structure on the data subset with only women who had at least two children.**
(DOC)

Table S6 **Factor loadings^ a^ from the pattern matrix for both the first and second data subsets.**
(DOC)
