# Supplementary material for: A Novel Quantitative Approach to Women’s Reproductive Strategies
Source: PLoS One. 2012 Oct 2;7(10):e46760. doi: 10.1371/journal.pone.0046760 (PMC3462799; doi:10.1371/journal.pone.0046760)
Supplement: Table S1 — Pattern matrix with rotated factor loadings for each variable in the six-factor structure on the data subset with no imputation of missing values. (DOC) [file pone.0046760.s001.doc]

**Table S1**: Pattern matrix with rotated factor loadings for each variable in the six-factor structure on the data subset with no imputation of missing values.

|  | **1** | **2** | **3** | **4** | **5** | **6** |
| --- | --- | --- | --- | --- | --- | --- |
|  | **Short-term mating strategy** | **Early onset of sexual activity** | **Reproductive output** | **Timing of childbearing** | **Breastfeeding** | **Child spacing** |
| *Age at first sexual intercourse* | -0.025 | **0.831** | -0.026 | 0.103 | -0.019 | -0.031 |
| *Number of sexual partners* | **0.622** | -0.369 | -0.134 | 0.180 | -0.018 | 0.001 |
| *Number of committed relationships* | **0.918** | -0.135 | 0.072 | 0.000 | -0.041 | -0.001 |
| *Average duration of relationships* | **-1.019** | -0.142 | 0.017 | 0.048 | -0.035 | -0.023 |
| *Number of pregnancies* | -0.039 | -0.113 | **0.789** | 0.078 | 0.048 | -0.031 |
| *Age at first birth* | 0.036 | 0.118 | -0.398 | **0.808** | 0.030 | -0.236 |
| *Age at last birth* | -0.008 | 0.064 | 0.284 | **0.909** | 0.024 | 0.231 |
| *Number of children* | -0.034 | 0.029 | **1.045** | -0.003 | 0.037 | -0.091 |
| *Average inter-birth interval* | 0.036 | -0.009 | -0.164 | 0.037 | 0.013 | **0.903** |
| *Ever breastfed* | 0.002 | -0.014 | -0.086 | -0.072 | **1.018** | -0.026 |
| *Duration of breastfeeding* | -0.011 | 0.002 | 0.135 | 0.097 | **0.918** | 0.043 |

χ^2^_(4)_ = 5.434, p = 0.246, n = 452

RMSEA = 0.028 (90% CI = 0.000 – 0.081), PCLOSE = 0.688

CFI = 1.000, TLI = 0.996

Factor loadings provide the direction and magnitude of the relationship between each variable and factor.

Bolding indicates factor loadings above |0.5|.
