# Supplementary material for: A Novel Quantitative Approach to Women’s Reproductive Strategies
Source: PLoS One. 2012 Oct 2;7(10):e46760. doi: 10.1371/journal.pone.0046760 (PMC3462799; doi:10.1371/journal.pone.0046760)
Supplement: Table S2 — Pattern matrix with rotated factor loadings for each variable in the six-factor structure on the data subset using average duration of breastfeeding per child rather than total duration of breastfeeding across all children. (DOC) [file pone.0046760.s002.doc]

**Table S2**: Pattern matrix with rotated factor loadings for each variable in the six-factor structure on the data subset using average duration of breastfeeding per child rather than total duration of breastfeeding across all children.

|  | **1** | **2** | **3** | **4** | **5** | **6** |
| --- | --- | --- | --- | --- | --- | --- |
|  | **Short-term mating strategy** | **Onset of sexual activity** | **Reproductive output** | **Timing of childbearing** | **Breastfeeding** | **Child spacing** |
| *Age at first sexual intercourse* | -0.037 | **-0.734** | -0.064 | 0.194 | -0.004 | -0.033 |
| *Number of sexual partners* | 0.485 | 0.485 | -0.147 | 0.202 | 0.016 | -0.001 |
| *Number of committed relationships* | **0.910** | 0.147 | 0.069 | -0.005 | -0.025 | 0.003 |
| *Average duration of relationships* | **-1.011** | 0.117 | 0.021 | 0.034 | -0.018 | -0.012 |
| *Number of pregnancies* | -0.013 | 0.183 | **0.805** | 0.120 | 0.016 | -0.044 |
| *Age at first birth* | 0.030 | -0.085 | -0.382 | **0.835** | 0.026 | -0.230 |
| *Age at last birth* | -0.001 | -0.063 | 0.322 | **0.906** | 0.022 | 0.241 |
| *Number of children* | -0.017 | -0.059 | **1.043** | -0.022 | 0.020 | -0.081 |
| *Average inter-birth interval* | 0.024 | 0.014 | -0.148 | 0.017 | 0.006 | **0.911** |
| *Ever breastfed* | -0.030 | 0.016 | 0.118 | -0.102 | **1.026** | -0.062 |
| *Average duration of breastfeeding* | 0.033 | -0.013 | -0.121 | 0.112 | **0.991** | 0.072 |

χ^2^_(4)_ = 7.724, p = 0.102, n = 718

RMSEA = 0.036 (90% CI = 0.000 – 0.074), PCLOSE = 0.678

CFI = 1.000, TLI = 0.993

Factor loadings provide the direction and magnitude of the relationship between each variable and factor.

Bolding shows factor loadings above |0.5|.
