# Supplementary material for: A Novel Quantitative Approach to Women’s Reproductive Strategies
Source: PLoS One. 2012 Oct 2;7(10):e46760. doi: 10.1371/journal.pone.0046760 (PMC3462799; doi:10.1371/journal.pone.0046760)
Supplement: Table S3 — Pattern matrix with rotated factor loadings for each variable in the six-factor structure on the data subset with only women who had natural menopause. (DOC) [file pone.0046760.s003.doc]

**Table S3**: Pattern matrix with rotated factor loadings for each variable in the six-factor structure on the data subset with only women who had natural menopause.

|  | **1** | **2** | **3** | **4** | **5** | **6** |
| --- | --- | --- | --- | --- | --- | --- |
|  | **Short-term mating strategy** | **Early onset of sexual activity** | **Reproductive output** | **Timing of childbearing** | **Breastfeeding** | **Child spacing** |
| *Age at first sexual intercourse* | -0.043 | **0.776** | -0.045 | 0.150 | 0.010 | -0.017 |
| *Number of sexual partners* | **0.585** | -0.381 | -0.164 | 0.194 | 0.014 | -0.005 |
| *Number of committed relationships* | **0.938** | -0.119 | 0.055 | 0.006 | -0.034 | 0.004 |
| *Average duration of relationships* | **-1.016** | -0.131 | 0.000 | 0.051 | -0.015 | -0.019 |
| *Number of pregnancies* | -0.002 | -0.189 | **0.757** | 0.148 | 0.069 | -0.046 |
| *Age at first birth* | 0.039 | 0.133 | -0.395 | **0.794** | 0.005 | -0.259 |
| *Age at last birth* | -0.011 | 0.061 | 0.264 | **0.925** | 0.016 | 0.234 |
| *Number of children* | -0.012 | 0.041 | **1.059** | -0.014 | 0.036 | -0.079 |
| *Average inter-birth interval* | 0.041 | -0.010 | -0.182 | 0.051 | 0.004 | **0.871** |
| *Ever breastfed* | -0.001 | -0.011 | -0.057 | -0.097 | **1.032** | -0.051 |
| *Duration of breastfeeding* | -0.004 | 0.015 | 0.079 | 0.110 | **0.966** | 0.064 |

χ^2^_(4)_ = 4.242, p = 0.374, n = 605

RMSEA = 0.010 (90% CI = 0.000 – 0.063), PCLOSE = 0.862

CFI = 1.000, TLI = 0.999

Factor loadings provide the direction and magnitude of the relationship between each variable and factor.

Bolding shows factor loadings above |0.5|.
