# Supplementary material for: A Novel Quantitative Approach to Women’s Reproductive Strategies
Source: PLoS One. 2012 Oct 2;7(10):e46760. doi: 10.1371/journal.pone.0046760 (PMC3462799; doi:10.1371/journal.pone.0046760)
Supplement: Table S4 — Pattern matrix with rotated factor loadings for each variable in the six-factor structure on the data subset with average inter-birth intervals for uniparous women set at the difference between their age at last birth and their age at menopause (or current age if pre-menopausal). (DOC) [file pone.0046760.s004.doc]

**Table S4**: Pattern matrix with rotated factor loadings for each variable in the six-factor structure on the data subset with average inter-birth intervals for uniparous women set at the difference between their age at last birth and their age at menopause (or current age if pre-menopausal).

|  | **1** | **2** | **3** | **4** | **5** | **6** |
| --- | --- | --- | --- | --- | --- | --- |
|  | **Short-term mating strategy** | **Early onset of sexual activity** | **Reproductive output** | **Timing of childbearing** | **Breastfeeding** | **Child spacing** |
| *Age at first sexual intercourse* | -0.031 | **0.759** | -0.057 | 0.170 | -0.013 | 0.011 |
| *Number of sexual partners* | 0.479 | -0.486 | -0.172 | 0.208 | 0.002 | 0.034 |
| *Number of committed relationships* | **0.906** | -0.152 | 0.065 | 0.003 | -0.043 | -0.012 |
| *Average duration of relationships* | **-1.012** | -0.116 | 0.020 | 0.036 | -0.029 | -0.019 |
| *Number of pregnancies* | -0.031 | -0.196 | **0.722** | 0.169 | 0.061 | -0.091 |
| *Age at first birth* | 0.031 | 0.116 | **-0.532** | **0.727** | 0.005 | -0.141 |
| *Age at last birth* | 0.000 | 0.050 | 0.242 | **0.924** | 0.028 | 0.067 |
| *Number of children* | -0.021 | 0.055 | **0.969** | 0.022 | 0.056 | -0.177 |
| *Average inter-birth interval* | 0.007 | 0.006 | -0.084 | 0.023 | 0.001 | **0.965** |
| *Ever breastfed* | -0.005 | -0.022 | -0.102 | -0.096 | **1.019** | -0.045 |
| *Duration of breastfeeding* | 0.007 | 0.020 | 0.140 | 0.121 | **0.939** | 0.059 |

χ^2^_(4)_ = 4.876, p = 0.300, n = 718

RMSEA = 0.017 (90% CI = 0.000 – 0.061), PCLOSE = 0.866

CFI = 1.000, TLI = 0.998)

Factor loadings provide the direction and magnitude of the relationship between each variable and factor.

Bolding indicates factor loadings above |0.5|.
