# Supplementary material for: A Novel Quantitative Approach to Women’s Reproductive Strategies
Source: PLoS One. 2012 Oct 2;7(10):e46760. doi: 10.1371/journal.pone.0046760 (PMC3462799; doi:10.1371/journal.pone.0046760)
Supplement: Table S5 — Pattern matrix with rotated factor loadings for each variable in the six-factor structure on the data subset with only women who had at least two children. (DOC) [file pone.0046760.s005.doc]

**Table S5**: Pattern matrix with rotated factor loadings for each variable in the six-factor structure on the data subset with only women who had at least two children.

|  | **1** | **2** | **3** | **4** | **5** | **6** |
| --- | --- | --- | --- | --- | --- | --- |
|  | **Short-term mating strategy** | **Onset of sexual activity** | **Reproductive output** | **Timing of childbearing** | **Breastfeeding** | **Child spacing** |
| *Age at first sexual intercourse* | -0.044 | **-0.689** | -0.045 | 0.261 | -0.009 | -0.039 |
| *Number of sexual partners* | 0.408 | **0.565** | -0.112 | 0.211 | -0.002 | -0.023 |
| *Number of committed relationships* | **0.897** | 0.147 | 0.057 | -0.028 | -0.041 | 0.013 |
| *Average duration of relationships* | **-1.015** | 0.093 | 0.021 | 0.007 | -0.028 | -0.009 |
| *Number of pregnancies* | -0.024 | 0.209 | **0.747** | 0.127 | 0.057 | -0.019 |
| *Age at first birth* | 0.026 | -0.083 | -0.316 | **0.851** | 0.013 | -0.278 |
| *Age at last birth* | -0.005 | -0.027 | 0.260 | **0.900** | 0.018 | 0.291 |
| *Number of children* | 0.003 | -0.059 | **1.119** | -0.031 | 0.022 | -0.063 |
| *Average inter-birth interval* | 0.032 | 0.010 | -0.147 | 0.025 | 0.009 | **0.914** |
| *Ever breastfed* | -0.006 | 0.019 | -0.077 | -0.083 | **1.008** | -0.040 |
| *Duration of breastfeeding* | 0.008 | -0.019 | 0.107 | 0.097 | **0.937** | 0.052 |

χ^2^_(4)_ = 4.343, p = 0.362, n = 644

RMSEA = 0.012 (90% CI = 0.000 – 0.062), PCLOSE = 0.870

CFI = 1.000, TLI = 0.999

Factor loadings provide the direction and magnitude of the relationship between each variable and factor.

Bolding shows factor loadings above |0.5|.
