# Supplementary material for: A Novel Quantitative Approach to Women’s Reproductive Strategies
Source: PLoS One. 2012 Oct 2;7(10):e46760. doi: 10.1371/journal.pone.0046760 (PMC3462799; doi:10.1371/journal.pone.0046760)
Supplement: Table S6 — Factor loadings a from the pattern matrix for both the first and second data subsets. (DOC) [file pone.0046760.s006.doc]

**Table S6:** Factor loadings ^a^ from the pattern matrix for both the first and second data subsets.

| **Factor** | **1** | | **2** | | **3** | | **4** | | **5** | | **6** | |
| --- | --- | --- | --- | --- | --- | --- | --- | --- | --- | --- | --- | --- |
| **Data subset** | ***1st^b^*** | ***2nd^c^*** | ***1st*** | ***2nd*** | ***1st*** | ***2nd*** | ***1st*** | ***2nd*** | ***1st*** | ***2nd*** | ***1st*** | ***2nd*** |
| *Age at first sexual intercourse* | -0.183 | 0.036 | **0.605** | **-0.845** | -0.063 | -0.049 | 0.202 | 0.178 | -0.048 | 0.007 | -0.015 | -0.045 |
| *Number of sexual partners* | **0.643** | 0.378 | -0.346 | **0.595** | -0.183 | -0.135 | 0.120 | 0.251 | 0.011 | 0.000 | -0.029 | -0.014 |
| *Number of committed relationships* | **0.984** | **0.809** | -0.054 | 0.269 | 0.093 | 0.060 | -0.007 | -0.014 | -0.034 | -0.053 | 0.007 | 0.005 |
| *Average duration of relationships* | **-0.979** | **-1.049** | -0.107 | 0.113 | 0.031 | 0.024 | 0.036 | 0.025 | -0.021 | -0.025 | -0.018 | -0.019 |
| *Number of pregnancies* | -0.024 | -0.012 | -0.293 | 0.116 | **0.718** | **0.795** | 0.159 | 0.107 | 0.036 | 0.075 | -0.035 | -0.04 |
| *Age at first birth* | 0.072 | -0.002 | 0.125 | -0.095 | -0.403 | -0.383 | **0.681** | **0.835** | -0.002 | 0.036 | -0.427 | -0.163 |
| *Age at last birth* | -0.006 | -0.008 | 0.026 | -0.052 | 0.164 | 0.333 | **1.029** | **0.880** | 0.015 | 0.018 | 0.142 | 0.210 |
| *Number of children* | -0.014 | -0.019 | 0.047 | -0.047 | **1.069** | **1.037** | 0.032 | -0.031 | 0.050 | 0.037 | -0.046 | -0.088 |
| *Average inter-birth interval* | 0.066 | 0.012 | 0.002 | 0.008 | -0.291 | -0.082 | 0.223 | 0.008 | 0.026 | 0.002 | **0.708** | **1.101** |
| *Ever breastfed* | -0.019 | -0.004 | -0.024 | 0.031 | -0.083 | -0.069 | -0.104 | -0.051 | **1.024** | **1.002** | -0.042 | -0.038 |
| *Duration of breastfeeding* | 0.017 | 0.009 | 0.028 | -0.025 | 0.124 | 0.100 | 0.131 | 0.052 | **0.943** | **0.943** | 0.054 | 0.051 |

^a^ When comparing the same factor from different models, one must examine the magnitude of the factor loadings and the consistency of the signs within a factor, rather than the signs of each variable across factors.

^b^ ‘1^st^’ refers to the first data subset the model was originally developed on.

^c^ ‘2^nd^’ refers to the second data subset the model validity was evaluated on.

Bolding shows factor loadings above |0.5|.
